# Effect of Ag Modification on the Structure and Photocatalytic Performance of TiO_2_/Muscovite Composites

**DOI:** 10.3390/molecules28073187

**Published:** 2023-04-03

**Authors:** Fengqiu Qin, Ling Zhang, Yuhao Luo, Lili He, Shiji Lu, Li Xu, Xiaodong Zhu, Wei Feng

**Affiliations:** 1 School of Mechanical Engineering, Chengdu University, Chengdu 610106, China; 2 College of Materials and Chemistry & Chemical Engineering, Chengdu University of Technology, Chengdu 610051, China

**Keywords:** TiO_2_, muscovite, Ag modification, photocatalytic activity

## Abstract

Ag/TiO_2_/muscovite (ATM) composites were prepared by the sol–gel method and the effects of Ag modification on the structure and photocatalytic performance were investigated. The photocatalysts were characterized using X-ray diffraction (XRD), scanning electron microscopy (SEM), transmission electron microscopy (TEM), Brunauer–Emmett–Teller surface area (BET), X-ray photoelectron spectroscopy (XPS), Fourier transform infrared spectra (FTIR), photoluminescence spectra (PL) and ultraviolet–visible diffuse reflectance spectra (DRS). The photocatalytic activity of the obtained composites was evaluated by taking 100 mL (10 mg/L) of Rhodamine B (RhB) aqueous solution as the target pollutant. The muscovite (Mus) loading releases the agglomeration of TiO_2_ particles and the specific surface area increases from 17.6 m^2^/g (pure TiO_2_) to 39.5 m^2^/g (TiO_2_/Mus). The first-order reaction rate constant increases from 0.0009 min^−1^ (pure TiO_2_) to 0.0074 min^−1^ (150%TiO_2_/Mus). Ag element exists in elemental silver. The specific surface area of 1-ATM further increases to 66.5 m^2^/g. Ag modification promotes the separation of photogenerated electrons and holes and increases the visible light absorption. 1%Ag-TiO_2_/Mus (1-ATM) exhibits the highest photocatalytic activity. After 100 min, the rhodamine B (RhB) degradation degrees of PT, 150%TiO_2_/Mus and 1-ATM are 10.4%, 48.6% and 90.6%, respectively. The first-order reaction rate constant of 1-ATM reaches 0.0225 min^−1^, which is 25 times higher than that of pure TiO_2_.

## 1. Introduction

Rhodamine B (RhB) is a typical dye organic compound which is primarily used for industrial dyeing. RhB possesses carcinogenicity and teratogenicity, causing certain harm to human health and the sustainable development of ecosystems when it is discharged into water bodies [1,2]. The methods of removing RhB include physical adsorption, chemical precipitation, biological filtration, etc. [3,4,5]. Because photocatalysts are able to degrade organic dyes into small inorganic molecules such as water and carbon dioxide directly under the irradiation of a light source without producing secondary pollution, this green technology has attracted wide attention [6,7,8,9]. Among numerous semiconductor photocatalyst materials, TiO_2_ has received the most extensive research due to its low cost, stable chemical properties, non-toxicity and harmlessness [10,11,12,13,14]. However, nanoscale TiO_2_ tends to readily aggregate, which results in a reduction in active reaction sites and adsorption performance. Using a matrix to load TiO_2_ can effectively reduce the agglomeration between particles, increasing the specific surface area and providing more reaction sites [15,16,17,18]. Muscovite (Mus) possesses the advantages of low price, acid and alkali resistance, heat resistance and chemical stability, and is commonly employed to load TiO_2_ to release its agglomeration [19,20,21,22]. Li [21] et al. prepared TiO_2_/Mus using a hydrothermal method and found that the particles’ agglomeration released after loading on Mus, which is beneficial to advancing photocatalytic performance.

On the other hand, the band gap width of TiO_2_ is large (3.2 eV), meaning it is only active in the ultraviolet light range with short wavelengths, limiting its applicability in the visible light range. In addition, the rapid recombination of photogenerated charges reduces photocatalytic activity [10,23]. Therefore, it is necessary to modify TiO_2_ to improve its photocatalytic performance. Ion doping, semiconductor coupling and noble metal decoration are common modification methods [24,25,26]. Feng et al. [14] prepared Fe/N co-doped nano-TiO_2_ using the solvothermal method and the degradation degree of RhB increased from 57.4% (pure TiO_2_) to 96.2% after irradiation for 60 min. The co-doping of Fe and N elements reduced the band gap width, which broadened the visible light response range, improving photocatalytic activity. Akhter et al. [25] synthesized ZnO/TiO_2_ composites using the sol–gel method and the degradation degree of MB reached 96% after illumination for 3 h. The combination of ZnO and TiO_2_ forms II-type semiconductor junctions which promote the separation of photogenerated electrons and holes, improving quantum utilization. Because the work functions of noble metals are larger than that of TiO_2_, when they make contact to form Schottky junctions, the photogenerated electrons generated by TiO_2_ are transferred to noble metals. Due to the existence of Schottky junctions, electrons in noble metals are prevented from flowing back into TiO_2_, which promotes the separation of photogenerated charges [27,28,29,30]. Additionally, owing to the surface plasmon resonance effect, noble metal modification can enhance the absorption in the visible light region, advancing the photocatalytic performance [31,32,33]. Bamola et al. [26] prepared Au-TiO_2_ using an inert gas evaporation method and it was found that Au and TiO_2_ formed Schottky junctions which favored the separation of photogenerated electrons and holes, inhibiting the recombination of carriers. Meanwhile, the absorption of composite materials in the visible light region was enhanced by Au decoration, which promoted the photocatalytic performance.

In this study, to combine the advantages of muscovite loading, which alleviates the agglomeration of TiO_2_ particles, and noble metal modification, which enhances the quantum utilization and visible light absorption simultaneously, Ag-modified and Mus-loaded TiO_2_ composite materials were prepared using a sol–gel method. The crystal structure, surface morphology, specific surface area, elemental composition, valence state and optical properties of the composite photocatalysts were analyzed with XRD, SEM, TEM, BET, XPS, FTIR, PL and RDS. The photocatalytic activity was evaluated by measuring the degradation degree of RhB and the mechanism of improving the photocatalytic performance with Mus loading and Ag modification was discussed.

## 2. Results and Discussion

### 2.1. Phase Composition

**Figure 1:**
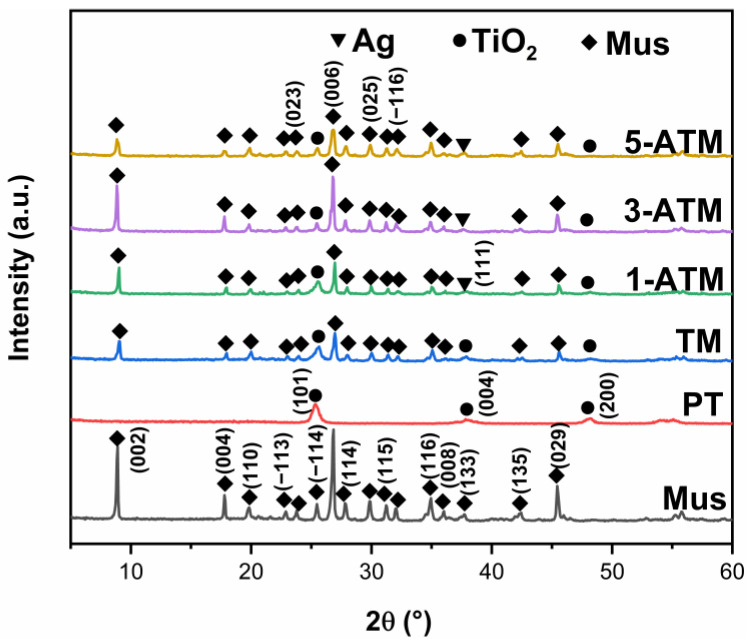
XRD patterns of samples.

Figure 1 shows the XRD patterns of pure TiO_2_ (PT), 150%TiO_2_/Mus (TM) and Ag/TiO_2_/muscovite (ATM). The three strong peaks in the XRD diffraction pattern of Mus appearing at 8.9°, 26.8° and 45.5° correspond to the (002), (006) and (029) crystal planes. The diffraction peaks of PT at 2θ = 25.3°, 37.8° and 48.1° correspond to the (101), (004) and (200) crystal planes of anatase, respectively, which means that TiO_2_ is single-phase anatase [32,34]. The diffraction peaks of anatase and Mus appear in the pattern of TM simultaneously, indicating that TiO_2_/Mus composites are formed. After Ag modification, diffraction peaks at 2θ = 38.1°, indexed to the (111) crystal plane of metallic Ag, can be detected, indicating that Ag/TiO_2_/muscovite composites are obtained [35,36]. With the increase in Ag content, the diffraction peak intensity of the (111) plane is enhanced. Figure S1 shows the XRD patterns of PT and TiO_2_/Mus. As the content of Mus increases from 15% to 200%, the intensity of the Mus diffraction peaks increases, while the intensity of the TiO_2_ diffraction peaks decreases and the half-height width expands gradually, implying that Mus loading increases the amorphous composition and decreases the crystallinity of TiO_2_.

### 2.2. Morphology and BET Surface Area

**Figure 2:**
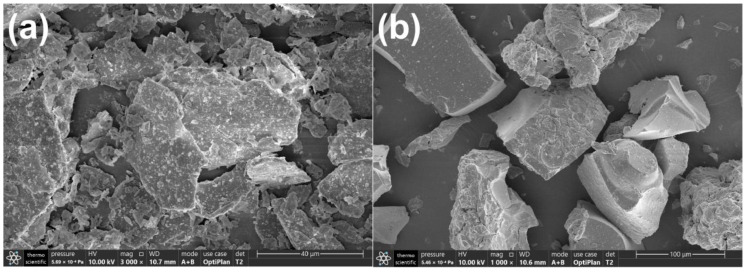

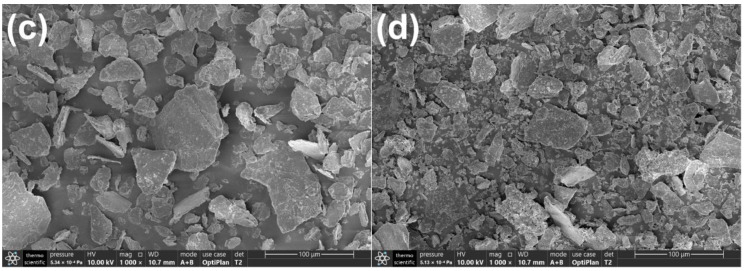
SEM images of Mus (**a**), PT (**b**), TM (**c**) and 1-ATM (**d**).

Figure 2 shows the SEM images of samples. As can be seen in Figure 2a, Mus particles present flaky shapes. It is observed in Figure 2b that PT prepared with the sol–gel method shows a significant agglomerative phenomenon, and the particle sizes are massive. When the Mus/TiO_2_ mass ratio is 150%, it can be observed in Figure 2c that TiO_2_ particles are evenly distributed on the surface of Mus, which reduces the aggregation of TiO_2_. Figure S2 shows the SEM images of 15%TiO_2_/Mus and 200%TiO_2_/Mus, from which it can be seen that with increasing Mus content, the agglomeration of TiO_2_ particles reduces more clearly. In Figure 2d, ATM shows comparable morphology to TM, which suggests that Ag modification does not cause the re-aggregation of TiO_2_ particles.

**Figure 3:**
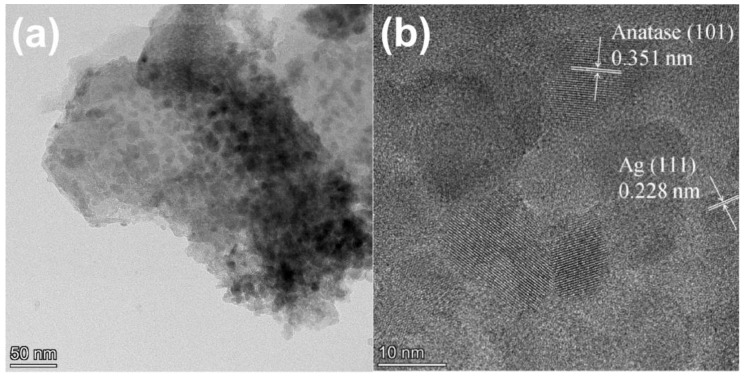
TEM (**a**) and HRTEM (**b**) images of 1-ATM.

Figure 3 shows the TEM and HRTEM images of 1-ATM. As can be seen in Figure 3a, TiO_2_ particles are dispersed on the Mus matrix and the size of a single particle ranges from 10 to 20 nm. Figure 3b shows the HRTEM image of 1-ATM. The marked crystal plane spacing of 0.351 nm is indexed to the anatase (101) crystal plane [21,37]. The lattice fringe of 0.228 nm can be ascribed to the crystal face of Ag (111) [38,39], indicating that Ag element exists as metallic Ag, which is consistent with the XRD results.

**Figure 4:**
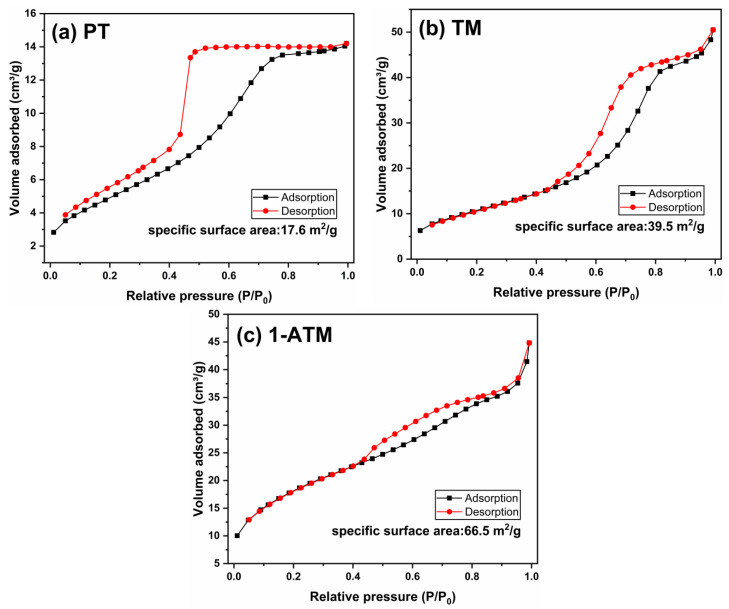
Nitrogen adsorption–desorption isotherms of PT (**a**), TM (**b**) and 1-ATM (**c**).

Figure 4 shows the N_2_ adsorption–desorption isotherms of PT, TM and 1-ATM. The specific surface area of PT is 17.6 m^2^/g. When it is loaded on Mus, the specific surface area increases to 39.5 m^2^/g (TM), indicating that the aggregation phenomenon of TiO_2_ particles is reduced after Mus loading. Ag modification further increases the specific surface area (66.5 m^2^/g) of 1-ATM. The increase in surface area provides more active sites for the photocatalytic reaction, which is beneficial to photocatalytic performance [15,16,22].

### 2.3. Element Valence State

**Figure 5:**
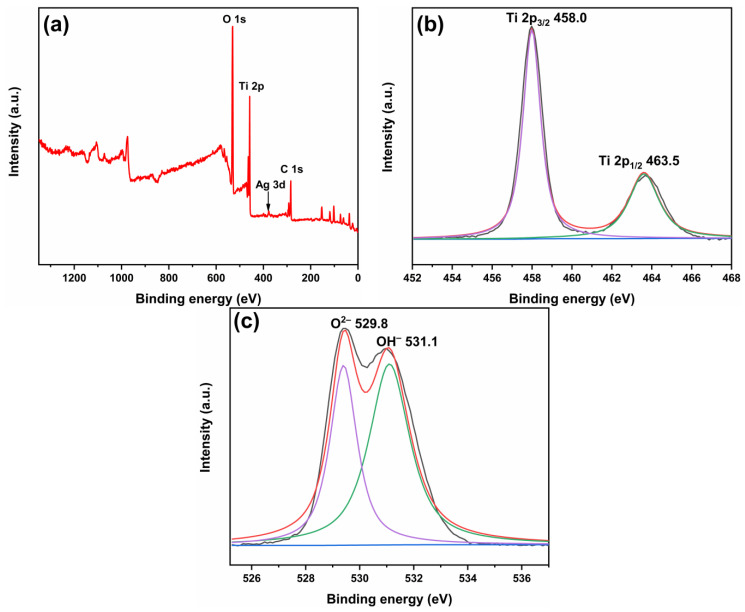
XPS spectra of 1-ATM: total spectrum (**a**), Ti 2p (**b**) and O 1s (**c**).

Figure 5 shows the XPS spectra of 1-ATM. The signal peaks of Ti, O and Ag elements appear in the total spectrum (Figure 5a). Figure 5b shows the high-resolution spectrum of Ti 2p. The two peaks at 458.0 and 463.5 eV are indexed to Ti 2p_3/2_ and Ti 2p_1/2_, indicating that Ti element exists in the form of 4+ [32,40,41]. The high-resolution spectrum of O 1s is shown in Figure 5c. The two peaks at 529.8 and 531.1 eV correspond to lattice oxygen (O^2−^) and surface hydroxyl (OH^−^) [42,43].

### 2.4. FTIR Analysis

**Figure 6:**
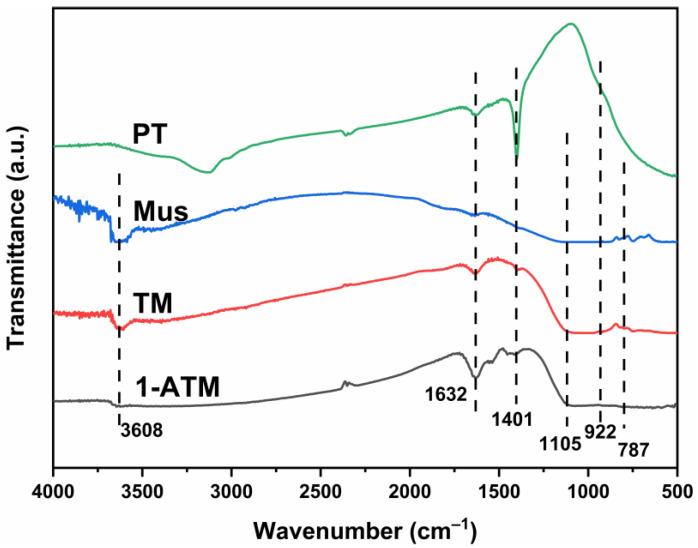
FTIR spectra of PT, Mus, TM and 1-ATM.

Figure 6 shows the FTIR spectra of PT, Mus, TM and 1-ATM. Mus shows a relatively obvious absorption band in the high-frequency region and the broad absorption band at 3608 cm^−1^ can be attributed to the Al-O-H stretching vibration [44]. The peak at 1632 cm^−1^ corresponds to the O-H bending vibration [32]. In addition, the wavelength of 1105 cm^−1^ corresponds to the asymmetric tensile vibration peak of Si-O-Si, indicating the existence of a SiO_2_ skeleton structure in Mus [45,46]. The weak peaks at 922 cm^−1^ are caused by the asymmetric tensile vibration of Si-O-Ti in Mus [45]. The absorption peak at 787 cm^−1^ corresponds to the vibration of the hydroxyl group of Mg-Al-OH in Mus, indicating that the structure of Mus has not been damaged by heat treatment. In PT, an obvious absorption peak is observed near wavelength 1401 cm^−1^, which is formed by the stretching mode of metal Ti ions and the absorption of atmospheric CO_2_ by carbonyl (C=O) [10]. Significantly, compared to Mus, the corresponding absorption peaks are also observed in TM and 1-ATM, which can be ascribed to the interaction between TiO_2_, Mus and Ag. There is no peak related to Ag that can be detected, which may be because Ag levels are too low for the instrument to detect.

### 2.5. Optical Property

**Figure 7:**
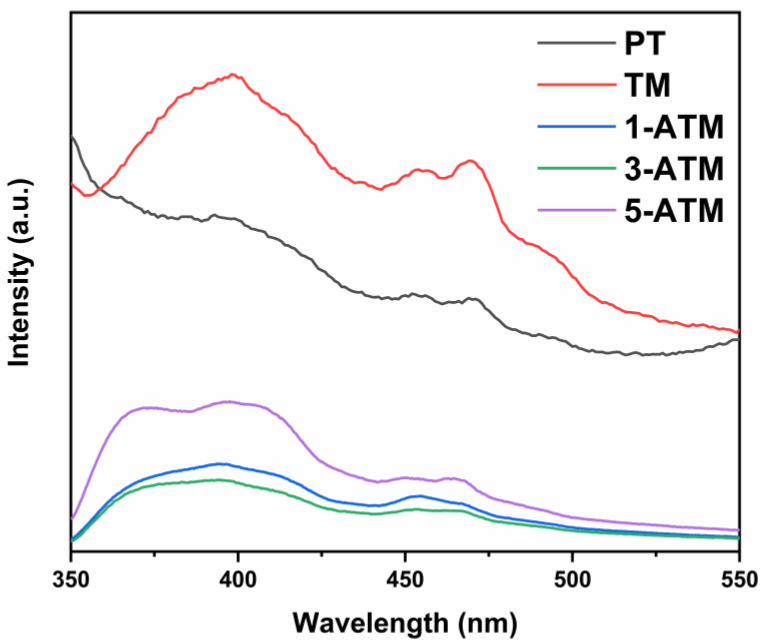
Photoluminescence spectra of PT, TM and ATM.

The separation of photogenerated electrons and holes is the core step of photodegradation. The higher the separation rate of photoinduced charge is, the more free radicals are generated during the degradation process, which is beneficial to the photocatalytic performance [36,42]. The photoluminescence (PL) peaks come from the released photons when the photogenerated electrons and holes recombine. Therefore, the lower PL peak intensity indicates a lower recombination of the photogenerated charges [47,48,49]. Figure S3 shows PL spectra of PT and TiO_2_/Mus in the wavelength range of 350–550 nm. It can be seen that the peak intensity of TiO_2_/Mus increases gradually when the loading amount of Mus increases from 15% to 200%, indicating that Mus loading aggravates the recombination of photogenerated charges. The XRD results show that the higher the Mus loading, the lower the TiO_2_ diffraction peak intensity, resulting in a decrease in TiO_2_ crystallinity and an increase in amorphous components and crystal defects which may become the recombination centers for photogenerated charges, thus increasing the PL peak intensity [22,50]. Figure 7 shows the PL spectra of PT, TM and ATM. After Ag modification, the recombination of photogenerated electrons and holes in ATM decreases compared to PT and TM. As the Fermi level of Ag is at the base of the TiO_2_ conduction band, the photogenerated electrons on the TiO_2_ conduction band transfer to the surface of Ag, which promotes the separation of photoinduced charges and improves quantum efficiency [35,51]. Significantly, when the concentration of Ag is excessive (5%), the PL peak intensity rises, which may be due to the formation of new recombination centers after superfluous Ag modification [43,52].

**Figure 8:**
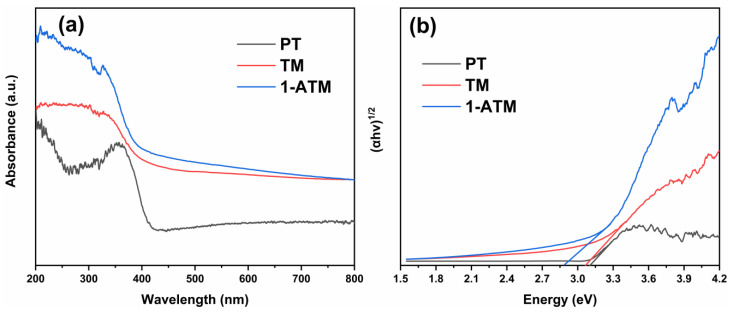
UV–visible absorption spectra (**a**) and band gap (**b**) of PT, TM and 1-ATM.

Figure 8 shows the UV–visible absorption spectra (a) and the band gap (b) of PT, TM and 1-ATM. As shown in Figure 8a, compared with PT, the absorption edge of TM and 1-ATM red shifts, indicating that Mus loading and Ag modification favor the utilization of visible light. The specific forbidden band width can be calculated as follows [52,53,54]:

*α*h*υ* = A(h*υ* − *Eg*)^1/*n*^ (1)

where h, υ, A and *Eg* are the Planck constant, the incident photon frequency, the proportional constant and the band gap (*Eg*), respectively. The value of n is related to the type of semiconductor, which is 2 for indirect semiconductors and is 1/2 for direct semiconductors [53,54]. Therefore, the value of *Eg* can be determined by the (*α*h*υ*)^1/2^-hυ curves in Figure 8b, which show that the band gaps of PT, TM and 1-ATM are 3.10, 3.06 and 2.89 eV, respectively. Ag modification diminishes the band gap of TiO_2_, reduces the energy barrier during the electron transition and promotes light absorption in the visible region.

### 2.6. Photocatalytic Performance

**Figure 9:**
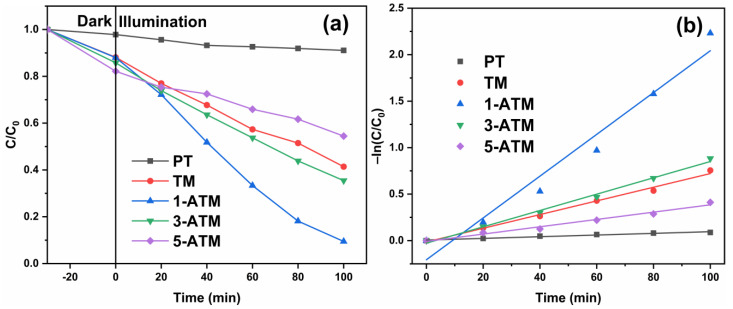
Degradation degree curves (**a**) and kinetics curves (**b**) of PT, TM and ATM.

Figure S4 presents the RhB degradation degrees of PT and TiO_2_/Mus, which show that TiO_2_/Mus exhibits the highest degradation degree when the Mus/TiO_2_ mass ratio is 150%. Figure 9a gives the RhB degradation degree curves of PT, TM and ATM. After 100 min, the degradation degrees of PT, TM, 1-ATM, 3-ATM and 5-ATM are 10.4%, 48.6%, 90.6%, 64.6% and 45.6%. Evidently, when TiO_2_ is loaded on Mus, the photocatalytic activity is significantly improved, which can be ascribed to the fact that the greater dispersion of TiO_2_ particles significantly enhances the specific surface area and provides more reactive sites. Moreover, Ag modification further advances the photocatalytic performance of TiO_2_/Mus composites. Combining PL and DRS spectra results, the enhancement of photocatalytic activity can be explained such that Ag modification reduces the recombination of photogenerated electrons and holes and enhances the absorption of visible light. The amount of Ag addition is the key to photocatalytic activity. ATM exhibits the highest photocatalytic activity when the Ag/Ti molar ratio is 1%. When the concentration of Ag is excessive, new recombination centers for photogenerated electrons and holes are formed, which is not conducive to the separation of photogenerated charges [43,52]. On the other hand, significant Ag deposition will cover the surface of TiO_2_ particles and hinder the absorption of the light source, reducing the photocatalytic degradation efficiency [39,55,56]. Figure 9b shows the kinetics curves of samples. The first-order reaction rate constants of PT, TM, 1-ATM, 3-ATM and 5-ATM are 0.0009, 0.0074, 0.0225, 0.0088 and 0.0039 min^−1^, respectively. 1-ATM produces the fastest reaction rate, which is in line with the degradation results.

**Figure 10:**
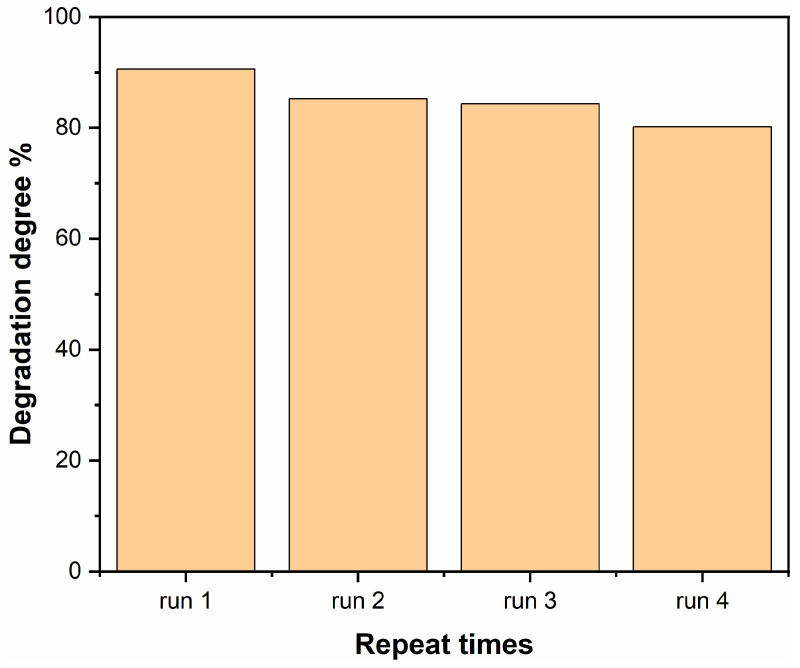
The reuse experiment of 1-ATM photocatalyst for RhB degradation.

To study the reusability of 1-ATM, the RhB degradation cycle experiment was carried out and the results are shown in Figure 10. After four cycles, the degradation degree of 1-ATM is 80.2%, which is slightly lower than 90.6%, indicating that 1-ATM has relatively considerable reusability.

**Figure 11:**
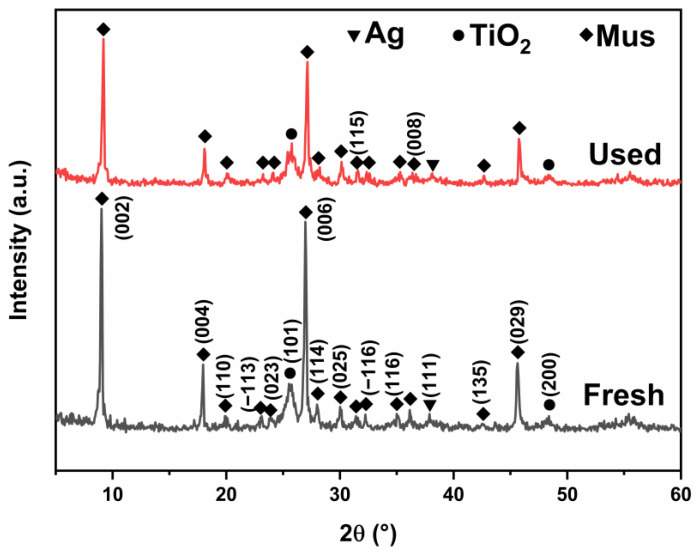
XRD patterns of 1-ATM photocatalyst before and after the photocatalytic experiment.

The XRD patterns of the fresh and used 1-ATM composite photocatalysts are shown in Figure 11. Compared to the initial sample, the positions of the diffraction peaks do not change and the peak intensities decrease marginally, which can be attributed to a small amount of undegraded RhB molecules remaining on the surface of 1-ATM [35].

**Figure 12:**
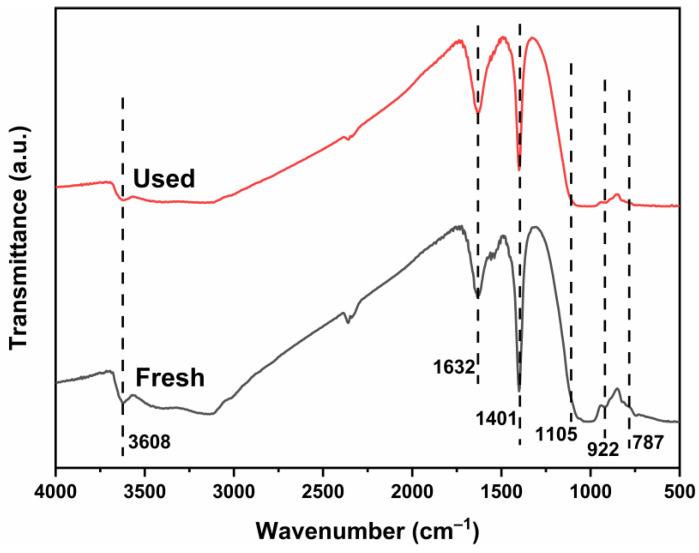
FTIR spectra of 1-ATM photocatalyst before and after the photocatalytic experiment.

To further verify the stability of 1-ATM, the FTIR spectra of the fresh and used 1-ATM are shown in Figure 12. Compared to the fresh spectrum, except for a slight decrease in peak strength, the characteristic peaks of the used sample can still be observed and have not shifted, indicating that the structure of the 1-ATM composite material is relatively stable.

### 2.7. Photocatalytic Degradation Mechanism

**Figure 13:**
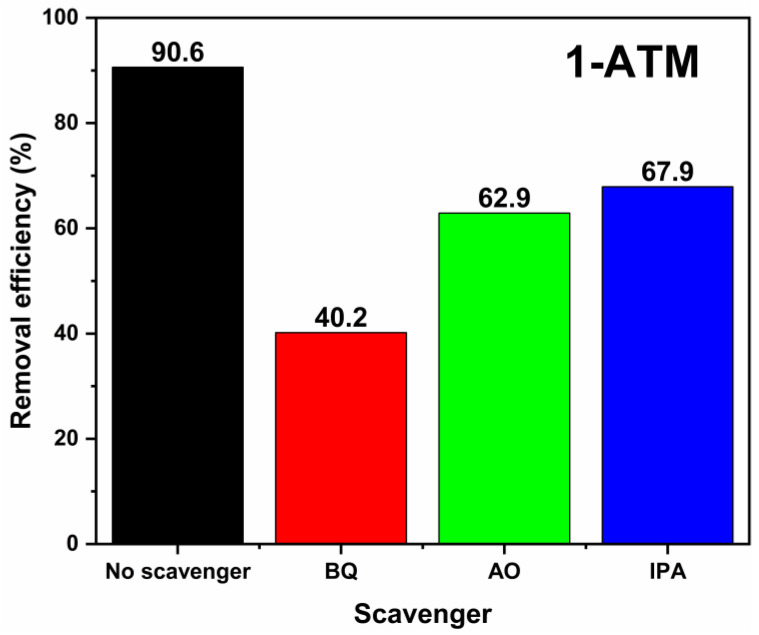
Degradation degrees of 1-ATM in the presence of different scavengers.

To determine the active species in the photodegradation process of 1-ATM, active species inhibition experiments were carried out on strong oxidizing groups such as h^+^, ·O_2_^−^ and ·OH, and the results are as shown in Figure 13. Based on the RhB degradation system, 2 mL of p-benzoquinone (BQ, ·O_2_^−^ trapping agent), isopropyl alcohol (IPA, ·OH trapping agent) or ammonium oxalate (AO, h^+^ trapping agent) was added to determine the active substances [57,58,59]. The degradation degrees of RhB are BQ (40.2%) < AO (62.9%) < IPA (67.9%) < no scavenger (90.6%), which indicates that ·O_2_^−^ is the main active group, while ·OH and h^+^ are secondary groups.

**Figure 14:**
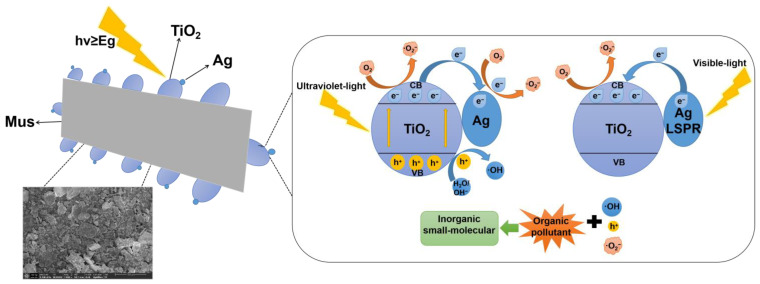
Schematic diagram of charge transfer and photodegradation of RhB for 1-ATM.

Using the characterization and photocatalytic degradation experiment results, the mechanism of photodegradation of RhB with the 1-ATM composite photocatalyst is proposed, as shown in Figure 14. Ag-TiO_2_ is firmly fixed on the surface of Mus using chemical bond links, which releases the agglomeration of TiO_2_ particles and provides more active reaction sites. When the photocatalysts are exposed to light irradiation in the ultraviolet region, electrons in the TiO_2_ valence band are excited to the conduction band, forming photogenerated electrons while leaving holes in the valence band. Photogenerated electrons with reducibility undergo a reduction reaction with O_2_ molecules adsorbed on the particle’s surface to generate ·O_2_^−^ radicals. Meanwhile, holes react with groups such as H_2_O and OH^−^ to generate ·OH radicals [3,25]. After Ag modification, as the work function of TiO_2_ is smaller than that of metal silver, electrons in TiO_2_ flow into metal Ag, while accumulating positive charges on TiO_2_. The accumulation of negative charges in metal Ag and positive charges in TiO_2_ creates a built-in electric field directed from TiO_2_ to metal Ag and causes the TiO_2_ energy band to bend upward, forming a Schottky barrier [27,30]. Electrons in Ag particles are prevented from flowing back to TiO_2_, owing to the existence of Schottky barriers. In the subsequent photocatalytic reaction, electrons accumulated on the metal Ag surface undergo a reduction reaction and holes accumulated in TiO_2_ undergo an oxidation reaction, leading to a new equilibrium state of the Fermi energy levels between metal Ag and TiO_2_ [29,30].

In the visible region, TiO_2_ valance band electrons cannot be excited. However, when metal Ag undergoes irradiation, a surface plasmon resonance effect occurs, causing free electrons to rise from their initial thermal equilibrium state to a higher energy state, increasing the energy of free electrons, which are then called hot electrons [30]. The yielded hot electrons will transfer to the TiO_2_ conduction band if their energies are greater than the conduction band potential of TiO_2_. This excitation mechanism does not need the photon energy to be greater than the TiO_2_ band gap, but only needs to meet the requirement of hυ ≥ E_f_ − E_c_ (E_c_ is the conduction band potential of TiO_2_ and E_f_ is the Fermi energy level when TiO_2_ and Ag reach an equilibrium state) [30]. In general, the necessary photon energy is smaller than the TiO_2_ band gap, making it more advantageous to use light sources for photocatalytic processes in the long wavelength range. Due to the existence of Schottky energy barriers, hot electrons that have been transferred to the TiO_2_ conduction band cannot flow back to the metal Ag [27,28,29,30]. When hot electrons are injected into the TiO_2_ conduction band, the corresponding thermal holes remain on Ag particles, causing an oxidation reaction.

## 3. Materials and Methods

### 3.1. Sample Preparation

Butyl titanate (C_16_H_36_O_4_Ti, AR, ≥98.0%), anhydrous ethanol (CH_3_CH_2_OH, AR, ≥99.7%), glacial acetic acid (C_2_H_4_O_2_, AR, ≥99.5%), silver nitrate (AgNO_3_, AR, ≥99.8%) and Rhodamine B (RhB) (C_28_H_31_N_2_O_3_Cl, AR, ≥99.0%) were purchased form Chengdu Kelon Chemical Reagent Factory (PR China).

Muscovite (Mus) acid treatment: First, 11.98 g Mus was placed in a beaker which was filled with 20 mL deionized water and 40 mL glacial acetic acid. The suspension was continuously stirred for 60 min and then washed repeatedly with deionized water and anhydrous ethanol. Finally, the sample was obtained for use after drying.

Pure TiO_2_: In total, 74 mL anhydrous ethanol and 34 mL tetrabutyl titanate were added into a beaker and stirred evenly to obtain solution A. Then, 8 mL deionized water, 7 mL glacial acetic acid and 37 mL anhydrous ethanol were mixed evenly to obtain solution B. A separating funnel was used to slowly drop solution B into solution A and stirring was maintained until sol was formed. After aging, the sol converted to gel, which was heat-treated at 450 °C for 1h in a muffle furnace to obtain TiO_2_. Pure TiO_2_ was labeled as PT.

TiO_2_/Mus: The acid-treated muscovite was added into the TiO_2_ sol under the condition of magnetic stirring and the other preparation steps were the same as above. The TiO_2_/Mus composite (Mus/TiO_2_ mass ratio = 150%) was labeled as TM.

Ag-TiO_2_/Mus: AgNO_3_ was added to solution B and the other preparation steps were the same to produce Ag-TiO_2_/Mus. The molar ratios of Ag/Ti were 1%, 3% and 5%. The Ag-TiO_2_/Mus samples were labeled as 1-ATM, 3-ATM and 5-ATM.

### 3.2. Sample Characterization

The crystal structure and composition of samples were analyzed using a DX-2700 X-ray diffractometer. Cu Kα radiation was used as an X-ray source, with a scanning range of 5–60° and a scanning speed of 0.06°/s (XRD, Dandong Haoyuan Instrument Co. Ltd., Dandong, China). The microscopic morphology of samples was studied with a Hitachi SU8220 scanning electron microscope (SEM) and a JEM-F200 transmission electron microscope (TEM and HRTEM) (FEI Company, Hillsboro, OR, USA). A V-sorb 2800S analyzer was used to measure the specific surface area (BET, Guoyi Precision Measurement Technology Co. Ltd., Beijing, China). The elements’ valence states were analyzed with XSAM800-type X-ray photoelectron spectroscopy (XPS, Thermo Scientific K-Alpha, Kratos Ltd., Manchester, UK). The photoluminescence spectra were measured with an F-4600 fluorescence spectrum analyzer with a Xe lamp at an excitation wavelength of 300 nm (PL, Shimadzu Group Company, Kyoto, Japan). The light absorption was analyzed with a UV-3600 UV–visible spectrophotometer (DRS, Shimadzu Group Company, Kyoto, Japan). An Agilent Cary630 Fourier transform infrared spectrometer was used to analyze the bonding condition (FTIR, Shanghai Weiyi Biotechnology Co. Ltd., Shanghai, China).

### 3.3. Photocatalysis Experiment

The photocatalytic activity of samples was evaluated by measuring the decomposition of RhB as a model pollutant. A combination of 100 mL (10 mg/L) RhB and 0.1 g sample was mixed in a beaker. The mixture was ultrasonically dispersed for 10 min and then stirred for 30 min under dark conditions to achieve the adsorption and desorption equilibrium. A xenon lamp was used as the light source and the samples were collected every 20 min. After centrifuging, the absorbance of the obtained solution was tested at the wavelength of 553 nm. The degradation degree of RhB was calculated using the formula (A_0_ − A_t_)/A_0_ × 100%, where A_0_ and A_t_ are the initial absorbance and t time absorbance.

## 4. Conclusions

TiO_2_/Mus composites were prepared using the sol–gel method and modified by Ag decoration. The agglomeration of TiO_2_ particles is reduced by Mus loading and the specific surface area is further increased after the addition of Ag, both of which provide more active sites for photocatalytic degradation reactions. Ag-TiO_2_ is firmly fixed on the surface of the Mus matrix by chemical bond links between TiO_2_ and Mus. ATM has much lower PL peak intensity than PT and Mus because Ag modification reduces the recombination of photogenerated charges. Ag modification also enhances the absorption in the visible light region. The degradation degree of RhB was employed to evaluate the photocatalytic performance of samples. The photocatalytic activity of 1-ATM is the highest and the degradation degree of RhB is 90.6% after illumination for 100 min, which is higher than that of pure TiO_2_ (10.4%). The first-order reaction rate constant of 1-ATM reaches 0.0225 min^−1^, which is 25 times higher than that of pure TiO_2_ (0.0009 min^−1^). The active species experiment using 1-ATM shows that ·O_2_^−^ radicals play a major role in the photodegradation process.

## Data Availability

Not applicable.

## Supplementary Materials

The following supporting information can be downloaded at www.mdpi.com/article/10.3390/molecules28073187/s1: Figure S1. XRD patterns of PT and TiO_2_/Mus; Figure S2. SEM images of 15%TiO_2_/Mus (a) and 200%TiO_2_/Mus (b); Figure S3. Photoluminescence spectra of PT and TiO_2_/Mus and Figure S4. Degradation degrees of PT and TiO_2_/Mus.
