# Supplementary material for: Effect of Ag Modification on the Structure and Photocatalytic Performance of TiO2/Muscovite Composites
Source: Molecules. 2023 Apr 3;28(7):3187. doi: 10.3390/molecules28073187 (PMC10095781; doi:10.3390/molecules28073187)
Supplement: Supplementary file 1 [file molecules-28-03187-s001.zip › molecules-2285935-supplementary.pdf]

molecules

Article

Effects of Ag Modification on the Structure and Photocatalytic Performance of TiO2/Muscovite Composites

Fengqiu Qin [1], Ling Zhang [1], Yuhao Luo [2], Lili He [1], Shiji Lu [1], Li Xu [1], Xiaodong Zhu [1,2]*, Wei Feng [1,]*

[1] School of Mechanical Engineering, Chengdu University; Chengdu 610106
[2] College of Materials and Chemistry & Chemical Engineering, Chengdu University of Technology; Chengdu 610051, China
*Correspondence: e-mail: xiaodangjia21@126.com (X.Z.); fengwei233@126.com (F.W.)

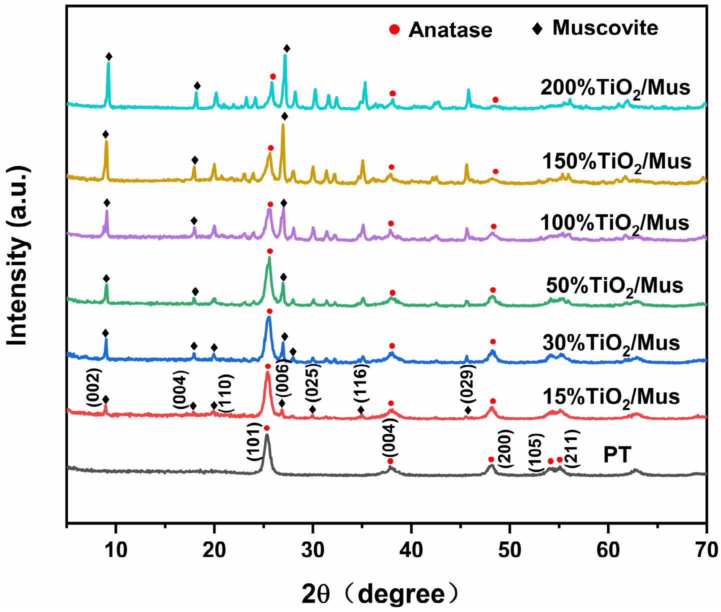

Figure S1. XRD patterns of PT and TiO_2/Mus.

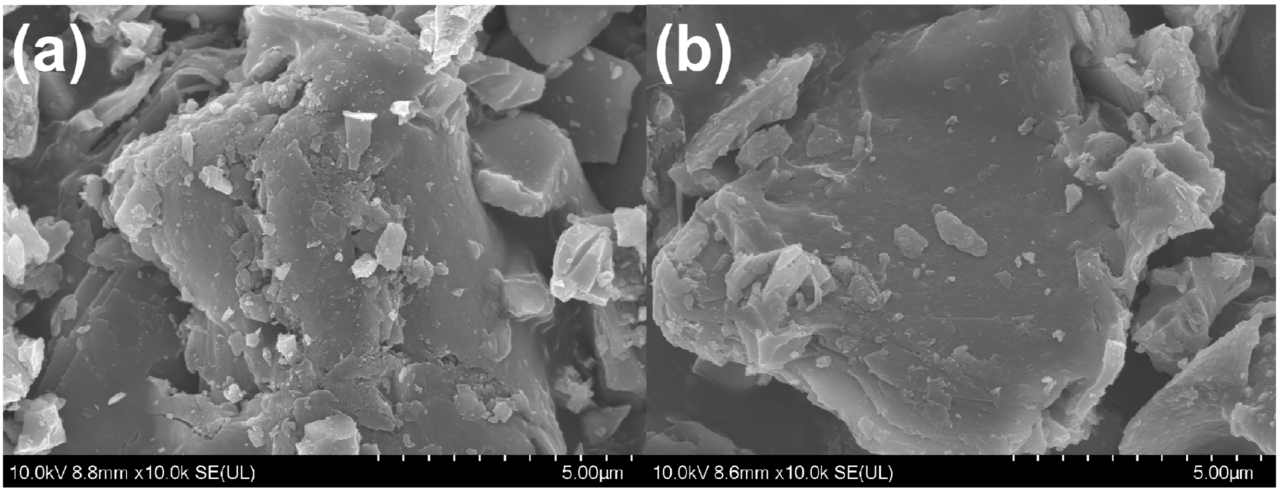

Figure S2. SEM images of 15%TiO_2/Mus (a) and 200%TiO_2/Mus (b).

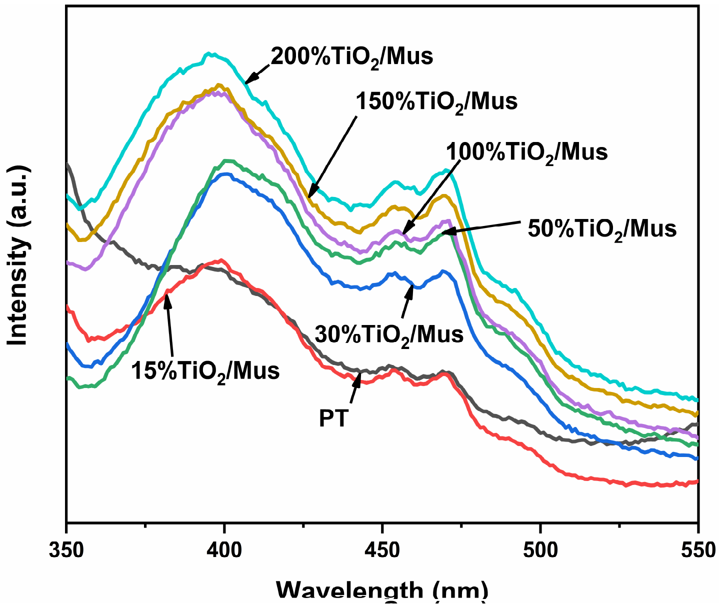

Figure S3. Photoluminescence spectra of PT and TiO_2/Mus.

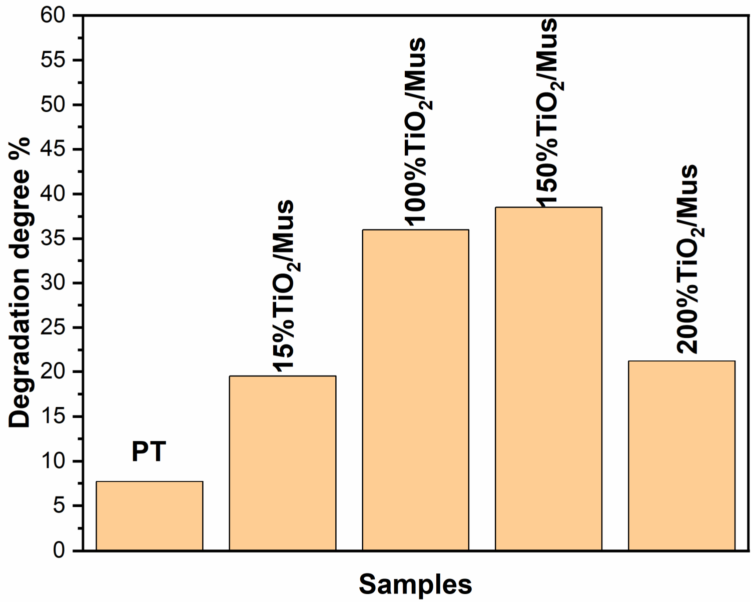

Figure S4. Degradation degrees of PT and TiO_2/Mus with difference mass ratio after 50 min irradiation.
